# Comprehensive review: types, clinical manifestations, diagnosis, and surgical management of ectopic gallbladder

**DOI:** 10.1097/JS9.0000000000003332

**Published:** 2025-09-02

**Authors:** Muhamad Zakaria Brimo Alsaman, Syed Muhammad Ali, Mohammad Nour Kitaz, Hala Sallah, Maya Sabboh, Rayan Badawi, Rana El Nahas, Ammar Albostani, Abdualh Fattal, Bashar Badawi, Mohannad Al-Tarakji

**Affiliations:** a Faculty of Medicine, University of Aleppo, Aleppo, Syria; b Acute Care Surgery, Hamad Medical Corporation, Doha, Qatar; c Clinical Surgery, Qatar University, Doha, Qatar; d Clinical Surgery, Weill Cornell Medical College in Qatar, Doha, Qatar; e Neurosurgery Department, Aleppo University Hospital, Aleppo, Syria; f Ophthalmology Department, Aleppo University Hospital, Aleppo, Syria; g Faculty of Medicine, Tishreen University, Latakia, Syria; h Neurology Department, Aleppo University Hospital, Aleppo, Syria; i Biotechnology, Faculty of Pharmacy and Food Sciences, University of Barcelona, Barcelona, Spain; j Department of Family Medicine and Community Health, University of Minnesota, Minneapolis, USA

**Keywords:** ectopic gallbladder, gallbladder, gastrointestinal tract, intrahepatic gallbladder, left-sided gallbladder, retroplaced gallbladder, suprahepatic gallbladder, transverse gallbladder

## Abstract

Ectopic gallbladder (EGB) is a rare condition where the gallbladder is located in an unusual position. EGB can be left-sided, intrahepatic, suprahepatic, transverse, or retroplaced. While often asymptomatic, EGB can present with abdominal pain, nausea, vomiting, or diarrhea. Our study aims to provide a comprehensive overview of EGB, outlining its different types, embryological origins, possible associated syndromes, available diagnostic approaches, and recommendations for diagnosis and surgical management. Our review identified a total of 122 case reports, 21 case series, 2 brief reports, and 4 letters to the editor, encompassing 206 patients with ectopic gallbladder. The dominant type of EGB was left-sided, accounting for 54.9% of cases. No apparent gender correlation was found. Most patients (81.6%) were symptomatic; only 18.4% were diagnosed incidentally. Abdominal pain and discomfort were the most common manifestations. EGB is typically symptomatic only when related gallbladder pathology occurs. Diagnosis cannot be reliably made via physical exam alone. While pre-operative diagnosis was made in 41.2 % of cases, 48.6% were diagnosed intraoperatively. Due to the limited sensitivity of ultrasound in detecting EGB variants, contrast-enhanced CT and MRCP became the preferred diagnostic tools for preoperative diagnosis due to their higher sensitivity. Laparoscopic cholecystectomy (LC) remains the optimal surgical approach, especially when advanced technologies are available. The majority of patients underwent laparoscopic surgery. Laparoscopic surgical approaches have emerged as the optimal surgical treatment.

## Introduction

Ectopic gallbladder (EGB) is a rare condition where the gallbladder is located in an unusual position rather than its normal anatomical place in the gallbladder fossa within segment 4b of the liver^[1,2]^. Recognizing EGB is important because it can pose a tricky challenge for surgeons pre and intraoperatively^[3]^. In addition, ectopic placement increases the chances of pathogenicity because of biliary stasis and torsion^[4]^.

**HIGHLIGHTS**

- Ectopic gallbladder is a rare anomaly that may occur due to either congenital factors or acquired causes.
- The most common types of aberrant gallbladders are left-sided, intrahepatic, suprahepatic, floating, retroplaced, and transverse.
- Abdominal pain and discomfort were the most common manifestations of ectopic gallbladder.
- The gold standard diagnostic tool for ectopic gallbladder is computed tomography (CT).
- Laparoscopic cholecystectomy emerges as the optimal surgical treatment for ectopic gallbladder diseases.

EGB has been reported in various abdominal areas; the most common locations include: under the left hepatic lobe, intrahepatic, transverse, retroplaced (retrohepatic or retroperitoneal), within the leaves of the lesser omentum or within the falciform ligament^[5,6]^.

EGB is often discovered when symptomatic, as it can present with abdominal discomfort, pain, nausea, and jaundice. Nevertheless, the abnormal location of the gallbladder is not related to its symptoms^[7]^. EGB can cause serious complications such as an increased risk of cholelithiasis, as well as torsion and herniation through the foramen of Winslow^[8]^. In the literature, almost half of EGB cases are discovered incidentally during surgeries performed for other causes^[9]^. Incidental discovery may be associated with an increased dissection surface, the possibility of injury to nearby organs, and increased operation time. Therefore, pre-operative diagnosis would be positively effective^[8]^.

Preoperative diagnostic tools include ultrasonography (US), computed tomography (CT), and magnetic resonance imaging (MRI)^[9]^. Intraoperative diagnostic techniques are also available when needed, such as fluorescent angiography^[10]^. Choices of treatment depend on the presence of symptoms and may entail observation or surgery, using either laparoscopic or open surgical approaches^[10]^.

This manuscript provides a comprehensive review of all accessible English-language case reports and case series studies that involved an ectopic gallbladder, summarizing key findings and tracing the evolution of its diagnosis and management. However, formal treatment guidelines cannot be established from the available data due to the rarity of the condition and the continuous evolution of diagnostic and surgical techniques. This article is compliant with the TITAN Guidelines 2025 – governing declaration and use of AI^[11]^.

## Methods

### Search strategy

We conducted an electronic database search in PubMed, Google Scholar, Scopus, and Web of Science (WoS) databases for ectopic gallbladder studies using the following search terms: “ectopic gallbladder” OR “intrahepatic gallbladder” OR “transverse gallbladder” OR “retroplaced gallbladder” OR “left sided gallbladder” OR “left-sided gallbladder” OR “dystopic retrohepatic gallbladder” OR “suprahepatic gallbladder” OR “floating gallbladder” OR “mesenteric gallbladder” OR “sinistroposition gallbladder” OR “retrorenal gallbladder” OR “gallbladder ectopia”.

### Study eligibility

We included all case reports and case series studies that involved an ectopic gallbladder. We aimed to analyze all individual cases of ectopic gallbladder to collect as much data as possible due to the uncommon nature of our study subject.

Exclusion criteria: We excluded cohort or case-control studies, conference abstracts, and reviews. As well as, studies that did not involve an ectopic gallbladder.

### Data collection

Data was extracted using Endnote X8 (Clarivate Inc.) and Microsoft Excel (Microsoft Office Inc.) to format the table. Four authors performed the data extraction. The extraction table included the authors’ names, year, paper design, and the number of patients, country, age, gender, gallbladder type, association with a syndrome, presentation, pain site, pain radiation, and other symptoms.

### Statistical analysis

Descriptive statistics were performed to calculate the number and percentage of participants for each sub-group. Statistical analysis was assessed by the Chi-Square test for each sub-group. *P* <0.05 was considered significant. One author performed the statistical analysis. Statistical analysis was done using SPSS version 26 (IBM Corp.).

### Artificial intelligence use

Artificial Intelligence was not used in the research or manuscript development.

## Results

**Figure 1.**
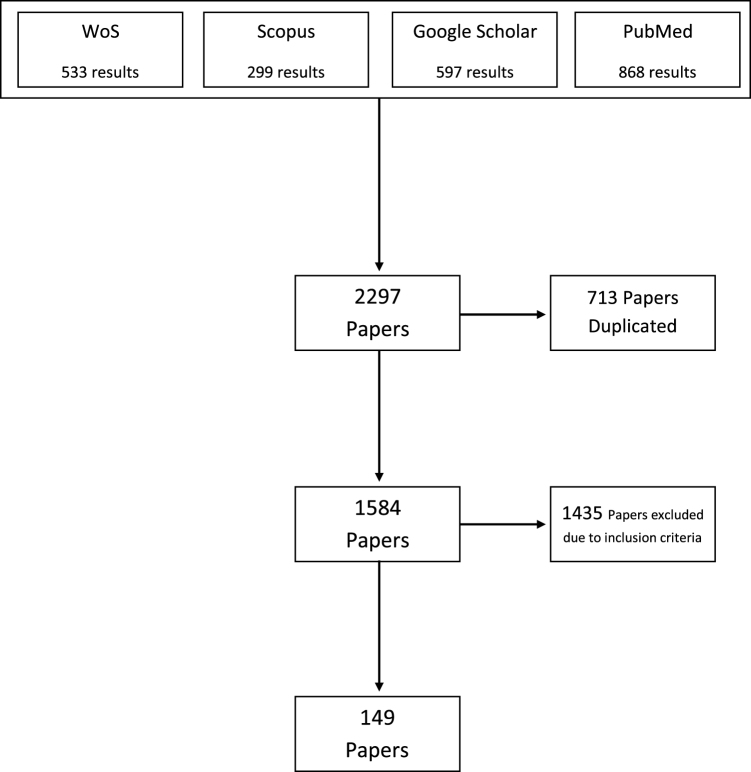
Flowchart diagram.

Our search, depicted in Fig. 1, across four databases (PubMed, Google Scholar, Scopus, and Web of Science) resulted in 2297 studies. Of these, 1435 studies were excluded because they did not meet our inclusion criteria, and 713 studies were excluded due to duplication. We included a total of 149 studies (case reports: 122, case series: 21, brief reports: 2, letters to the editor: 4). The search process was conducted to identify all published studies between 1936 and 2022. The full list of references included in the analysis is provided as supplementary material (Supplementary Digital Content File 1, available at: http://links.lww.com/JS9/E983).

**Figure 2.**
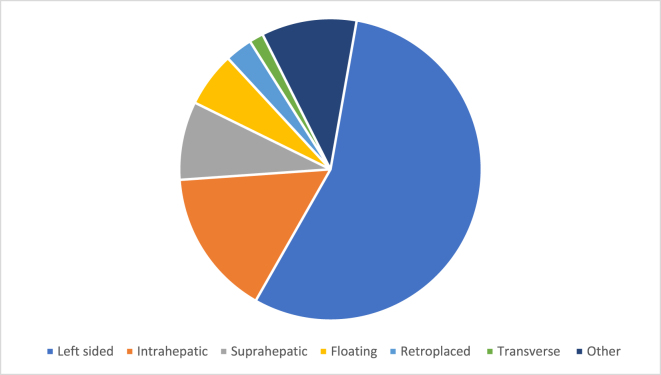
Types of ectopic gallbladder.

Six major categories of gallbladder malposition were identified. The most common type was the left-sided gallbladder, observed in 54.9% of cases. The intrahepatic gallbladder followed with 15.5%, while the transverse type was the least common, accounting for only 1.5% (Table 1). Figure 2 shows the distribution of different types of ectopic gallbladder according to their anatomical location. There was no gender difference (Table 1). EGB was also reported in presence of other syndromes as seen in 4 cases: 2 cases were with Mirizzi syndrome, one with Prune belly syndrome (PBS), and one with Churg–Strauss syndrome (Table 1).

**Table 1:** Clinical and demographic characteristics of patients with variant gallbladder types

Total (N)	206	
	Number	Percentage
Sex		
Male	103	50%
Female	103	50%
Gallbladder type		
Left-sided	113	54.9%
Intrahepatic	32	15.5%
Suprahepatic	17	8.3%
Floating	12	5.8%
Retroplaced	6	2.9%
Transverse	3	1.5%
Other	21	10.1%
Association with syndromes (N)		
Mirrizi	2	1%
Churg–Strauss	1	0.5%
Prune belly	1	0.5%
Presentation		
Incidental	38	18.4%
Symptomatic	168	81.6%

Symptomatic presentation was noted in 81.6% of cases. Abdominal pain was the most prevalent symptom, occurring in 35.6% of patients, most commonly in the right upper quadrant. In 22.8%, pain was localized to the epigastric region. Radiation of pain to the back, shoulder, or chest was occasionally reported. Other symptoms included nausea (20%), vomiting (16.3%), fever (12.7%), and, less frequently, anorexia, bloating, or diarrhea (Table 2).

**Table 2:** Main symptoms of ectopic gallbladder

	Number	Percentage*
The pain site		
Right upper	49	35.6%
Epigastric	32	22.8%
Periumbilical	1	0.7%
Diffuse	7	5.1%
Pain radiation		
Back	11	7.4%
R shoulder	6	4.4%
L shoulder	1	0.7%
R flank	4	3%
Chest	2	1.5%
Other symptoms		
Nausea	27	20%
Vomiting	22	16.3%
Anorexia	4	3%
Diarrhea	3	2.2%
Constipation	4	3%
Bloating	4	3%
Fevers	17	12.7%

^*^ Valid percentage.

As for diagnostic tools, 41.2 % of patients were diagnosed using pre-operative diagnostic tools. The most used pre-operative tool was contrast-enhanced CT in 76.5% of cases, followed by US 34.1%, and MRI 15.3%. 48.6% of patients were diagnosed intraoperatively, among those diagnosed during surgery, 55.9% underwent laparoscopic cholecystectomy, while 44.1% underwent open cholecystectomy (Table 3).

**Table 3:** Diagnostic tools of ectopic gallbladder

	Number	Percentage*
Pre-operative diagnosis tool		
Yes	85	41.2 %
Diagnosis tool		
US	29	34.1%
MRI	13	15.3%
CT	65	76.5%
Intraoperative diagnosis		
Yes	89	48.6%
Cholecystectomy (N = 145)		
Open	64	44.1%
Laparoscopic	81	55.9%

^*^ Valid percentage.

Of the 13 patients (18.4%) with incidentally discovered ectopic gallbladder, 46.2% underwent laparoscopic cholecystectomy, and 53.8% had open cholecystectomy. In contrast, among the 131 symptomatic patients (81.6%), 57.3% were managed laparoscopically, and 42.7% underwent open surgery (Table 4). Notably, 11.95% of patients initially scheduled for laparoscopic cholecystectomy (LC) required conversion to open surgery.

**Table 4:** Surgical approaches in patients with incidental and symptomatic gallbladder conditions

	N	Laparoscopic	Open surgery
Incidental	13	6 (46.2%)	7 (53.8%)
Symptomatic	131	75 (57.3%)	56 (42.7%)

Among the 48 patients who were pre-operatively diagnosed, 62.5% of them underwent LC and 37.5% underwent open surgery. While the 82 patients who were intraoperatively diagnosed, 53.7% of them underwent a laparoscopic procedure and 46.3% underwent open surgery. Based on these findings (Table 5), laparoscopic cholecystectomy remains the gold standard for the surgical management of ectopic gallbladder. Nevertheless, conversion to open surgery may be necessary in cases of unclear anatomy, technical difficulty, or limited laparoscopic expertise.

**Table 5:** Summary table that categorizes the types of ectopic gallbladder along with the surgical techniques performed for each type


Gallbladder type	Open	Laparoscopic
Left sided	21 (24.7%)	64 (75.3%)
Intrahepatic	17 (77.3%)	5 (22.7%)
Suprahepatic	10 (83.3%)	2 (16.7%)
Floating	9 (75%)	3 (25%)
Retroplaced	50.0%	50.0%
Transverse	0.0%	0.0%
Other	5 (62.5%)	3 (37.5%)

On the diagnostic side (Table 6), identifying ectopic gallbladder preoperatively remains challenging, as most cases are discovered intraoperatively. MRI appears to be the most effective diagnostic tool for ectopic gallbladder, though its availability and cost may pose limitations. Additionally, incomplete preoperative investigations often hinder diagnosis. While MRI is not suitable as the gold standard due to the low incidence of ectopic gallbladder, it is highly recommended in cases of diagnostic uncertainty.

**Table 6:** Summary table that categorizes the types of ectopic gallbladder along with the diagnostic techniques performed for each type

Gallbladder type	Diagnosis tool				
	No diagnosis tool	US	CT scan	MRI	More than one tool
Left sided	55.9%	8.6%	2.2%	24.7%	8.6%
Intrahepatic	62.5%	4.2%	0.0%	20.8%	12.5%
Suprahepatic	50.0%	0.0%	6.3%	18.8%	25.0%
Floating	72.7%	0.0%	0.0%	18.2%	9.1%
Retroplaced	0.0%	0.0%	0.0%	66.7%	33.3%
Transverse	66.7%	0.0%	0.0%	33.3%	0.0%
Other	52.6%	15.8%	5.3%	10.5%	15.8%

As for the transverse ectopic gallbladder, representing only 1.5% of cases, its inclusion remains debatable. While it may not add significant analytical value, retaining it might provide a more comprehensive classification spectrum.

## Discussion

Typically, the gallbladder is situated in the right upper quadrant below the right lobe of the liver, precisely in the gallbladder fossa between hepatic segments IV and V. Malposition, or aberrant situation of the gallbladder, can be either congenital or acquired^[2]^. Acquired cases are mostly secondary to changes distorting the liver topography and its relation to adjacent structures, such as cirrhosis and large nodules^[12]^.

### Historical milestones

To better understand the evolution of ectopic gallbladder diagnosis and management, we compiled key milestones from the literature (Table 7). This historical summary highlights the shift from incidental findings during open surgeries to advanced preoperative imaging and minimally invasive techniques, including robotic and ICG-guided cholecystectomy. These developments underscore the progressive refinement of surgical strategies in anatomically complex cases.

**Table 7:** Key historical milestones in ectopic gallbladder management

Author	Year	Milestone
Hochstetter^[13]^	1886	First anatomic description of an ectopic (left-sided) gallbladder.
Kehr^[14]^	1902	First surgical identification of an ectopic gallbladder.
Chung *et al*^[15]^	1997	First laparoscopic cholecystectomy on an ectopic gallbladder.
Ishizawa *et al*^[16]^	2009	Introduction of ICG fluorescent cholangiography in laparoscopy.
Gangemi *et al*^[10]^	2019	First robotic cholecystectomy for an ectopic gallbladder.

### Embryological development

Normal gallbladder development begins early in the fourth week of embryogenesis. The ventral part of the distal foregut thickens, forming the hepatic diverticulum which is the precursor of the liver, biliary duct system, and gallbladder. The hepatic diverticulum consists of three portions: the cranial bud (pars hepatica) develops into the liver, the ventral portion forms the head of the pancreas, whereas the caudal bud (pars cystica) gives rise to the gallbladder and extrahepatic biliary tract^[17]^. Ectopic placement of the gallbladder mostly results from developmental arrest in the migration of the gallbladder from reaching its normal superficial position in the second month of gestation. Several anomalies of the gallbladder are also reported such as agenesis, duplication, and septation^[18]^. An ectopic gallbladder can also accompany other anomalies such as portal venous anomalies (PVA). However, Yin *et al* concluded that the gallbladder’s location can’t always predict said anomalies. They alternatively suggested that a right-sided ligamentum teres (RSLT) is a more accurate predictor for PVAs, with the rationale that RSLT doesn’t always coexist with left-sided gallbladder (LSGB)^[19]^.

### Types of ectopic gallbladders

The six most common types of aberrant gallbladders in descending order of their prevalence are left-sided, intrahepatic, suprahepatic, floating, retroplaced, and transverse. There are other less common positions, like the falciform ligament^[20]^, extraperitoneal or subcutaneous^[21]^, hepatoduodenal ligament^[22]^, behind the pancreas^[23]^, embedded in the mesocolon^[24]^, retrocolic retroduodenal subhepatic^[25]^, and extra-abdominal^[26]^. In 2009 a new unexpected place for the gallbladder was discovered out of the biliary tree superior to the transverse colon, and it occurs only in two reported cases^[27]^.

The left-sided gallbladder (LSGB) or the Levo position, which is the most prevalent, is one where the gallbladder is situated on the under-surface of the left lobe of the liver to the left of round and falciform ligaments as defined by Gross^[28]^.

It has three types:

- Situs inversus which is a congenital malformation in which thoracic and/or abdominal organs are transposed in a mirror-image position^[29]^. Multiple factors may play into the etiology of Situs Inversus including genetics, an abnormal relationship of the embryo and the chorion, an abnormal relationship of the embryo and the umbilical vessels, a reversed spiral twist in the umbilical cord, as well as temperature, growth, and nutrition^[30]^. Situs inversus is not a pathologic state, nor does it predispose to a specific ailment. However, it is associated with several congenital anomalies in numbers far greater than in the normal population, these anomalies include left-sided gallbladder^[31]^, polysplenia, hypogenesis of the celiac artery, interruption of the inferior vena cava with continuation of the hemiazygos vein, and a preduodenal portal vein^[7]^.
- The right left-sided gallbladder or “false” left-sided gallbladder is an anomaly where the gallbladder is in its normal position but there is a right-sided round ligament/ligamentum teres. It was previously diagnosed as a left-sided gallbladder. However, Nagai *et al* proposed a modified classification, that it is an entirely different diagnosis since it doesn’t fit Gross’s definition^[32]^.
- True left-sided gallbladder, there have been three theories about its origin. First, the gallbladder might arise from the hepatic diverticulum per usual, however, then it migrates to the left lobe passing the falciform ligament. Second, an additional gallbladder might originate from the left hepatic duct with the original gallbladder failing to develop or atrophying^[28]^.Third, if the quadrate lobe fails to develop, that might result in a left-sided gallbladder^[33]^.

An intrahepatic gallbladder lies in a subcapsular position within the right lobe completely or partially surrounded by hepatic parenchyma. At first glance, the vesicle may seem absent^[28,34]^. During normal embryogenesis the gallbladder is intrahepatic but then it becomes extrahepatic. Therefore, an intrahepatic gallbladder in adults may be misdiagnosed as a normal-located gallbladder. An intrahepatic gallbladder could also be acquired after chronic inflammation^[35]^.

A suprahepatic gallbladder is often associated with a smaller-than-usual right lobe due to congenital or acquired reasons such as cirrhosis, vascular injury, or cancer^[36]^. However, there has been a report of Suprahepatic gallbladder accompanying hepatomegaly^[37]^. Suprahepatic gallbladders have been also seen in cases of diaphragmatic eventration, inversion of the liver, and abnormally mobile gallbladders^[34,36,38]^. Chilaiditi’s sign is a radiological sign that is reported in association with suprahepatic gallbladder. This sign is related to the interposition of the small bowel or the colon between the liver and the diaphragm, and it is associated with an abnormal development of the right liver lobe such as agenesis, hypoplasia or atrophy^[39]^.

A floating gallbladder is either: fully surrounded by the peritoneum, suspended from the liver by a mesentery, or the mesentery may not exist, and the cystic duct and artery are the only attachment^[38]^. This anomaly is a result of unusual migration of the pars cystica from the hepatic diverticulum during embryogenesis^[40]^.

A retroplaced gallbladder courses superiorly and posteriorly and lies under the inferior and posterior surfaces of the right lobe of the liver^[41]^. It can either be retrohepatic, retroperitoneal^[20]^, or retrorenal^[42]^. Mattone *et al* found that a retrohepatic gallbladder is often associated with hypoplasia, atrophy, or agenesis of the right lobe of the liver^[43]^.

A transverse gallbladder is one situated in a horizontal position inside the transverse fissure, and it is further back than usual^[41]^. This anomaly is possibly caused by abnormal migration or rotation of the definitive organ^[28]^.

### Clinical manifestations

EGB is typically asymptomatic and only becomes evident during disease or complications^[4,7]^. This explains why most reported cases (81.6%) were symptomatic. To clarify further, the majority of cases documented in the literature are symptomatic, likely due to publication bias, as asymptomatic cases are less commonly reported. Whereas incidental diagnosis accounted for only 18.4% of cases where patients presented to healthcare centers for illnesses unrelated to gallbladder disease manifestations.

EGB might develop the same pathologies as orthotopic gallbladder, such as cholelithiasis, cholecystitis, polyps, and empyema with similar manifisations^[44]^. According to Almas *et al*, approximately 75% of patients with left-sided gallbladder (LSGB) suffer from gallbladder-related diseases, such as cholecystitis, exhibiting the typical symptoms^[45]^. Despite the malposition of EGB, the neural supply and innervation remain unaffected since the nervous system does not undergo transposition, causing no change in symptoms.

Our results are consistent with the idea that EGB diseases present similarly to their counterparts in normally placed gallbladders, as the most common symptoms included right hypochondriac discomfort in 35.6% of cases, which radiates mostly to the back in 7.4% of cases. In addition, epigastralgia was present in 22.8% of cases, and nausea in 20% of them. Generally, symptoms are not related to the anomalous position of the gallbladder, but rather are largely dependent on the associated diseases^[7]^. For instance, EGB cholecystitis presents as colicky pain in the right subcostal region^[44]^. On the other hand, complications may arise from EGBs’ abnormal location^[4,7]^. Reported complications mainly include an increased risk of cholelithiasis due to bile stasis, mesenteric torsion, and, rarely, herniation through the Winslow foramen^[4]^.

### Diagnostic investigations

Accurate preoperative diagnosis and localization of EGB are critical since EGB may pose a challenge when discovered intraoperatively without proper planning from the surgeon. For instance, Pereira *et al* found that LSGB is associated with a higher probability of injuring the common bile duct due to poor dissection technique^[3]^. Therefore, careful preoperative radiological studies are essential to identify the ectopic gallbladder and alert the surgeon to atypical positioning. This enables mental readiness, modified trocar positioning, and the evaluation of additional methods such as ICG fluorescence or intraoperative cholangiography to reduce confusion during surgery and the risk of injury.

Conventional US is the first-line imaging modality used in most cases, considering its wide availability and good sensitivity in excluding gallstones and acute cholecystitis. It has a sensitivity of more than 90% for the diagnosis of acute calculous cholecystitis, presenting a thickened gallbladder wall and US Murphy sign^[7]^. However, the sensitivity of US is low in detecting bile duct anatomical variants and may occasionally lead to a false diagnosis^[7]^. Several causes were proposed to be the reason for the US undetectability of EGB, such as gallbladder-gastrointestinal fistulas or gallbladder atrophy due to filling with gallstones^[7]^. Moreover, even in ectopic gallbladders, the cystic duct and vascular supply of the gallbladder are usually normal. In fact, in all patients with LSGB although the whole course of the cystic duct is not visualized, the narrow neck of the gallbladder is clearly detected by preoperative US, in the frontolateral aspect of the main portal vein, which seems to be the key finding of this anomaly, leading to the diagnosis. True LSGB cases may appear as a cystic mass close to the left lobe of the liver or a mass with a narrow-connected neck to the bile duct in front of the pancreas^[41,43]^. Thus, when examining patients by US, if the gallbladder is not detectable in the usual location and there is a cystic mass near the left lobe of the liver, the right side must be carefully examined by US, which leads to or at least increases the chance of diagnosing left-side gallbladder^[41]^.

In some patients with suprahepatic gallbladder, the anomalous position is the result of a hepatic deformity due to macronodular liver cirrhosis. In such patients, unusual US findings of the gallbladder are sometimes problematic. It can occasionally appear as perihepatic ascites. In this circumstance, careful scanning should preclude a false diagnosis of ascites, but contrast-enhanced CT can more easily establish the diagnosis in patients with a markedly atrophied liver. Clinically, this pseudo-ascites should not be confused with true ascites to avoid a potentially lethal and unnecessary aspiration^[7]^.

Velimezis *et al* mentioned that useful tools to diagnose LSGB in the preoperative phase are drip infusion cholangiographic CT and magnetic resonance cholangiopancreatography (MRCP)^[44]^. contrast-enhanced CT scans and MRCP are considered the gold standard for EGB diagnosis due to their high sensitivity, but they are not routinely used for typical gallbladder problems^[4,46]^. However, these imaging modalities are highly beneficial in the preoperative assessment of EGB by confirming gallbladder anomalies and visualizing the bile duct anatomy and its connection to the gallbladder^[45,47]^.

In most cases, contrast-enhanced CT and MRCP provide sufficient information. However, some limitations exist, and additional investigations like Endoscopic retrograde cholangiopancreatography (ERCP), CT angiography, or intraoperative US might be needed in specific situations, particularly if the surgeon is unfamiliar with EGB^[44,48]^. MRCP is the commonly used modality of choice to confirm the diagnosis and identify any other possible abnormalities of the biliary tree and liver. MRI, as well as radionuclide imaging, are modalities used to demonstrate intrahepatic gallbladder^[49]^.

According to our findings, abdominal contrast-enhanced CT scans effectively localize the gallbladder within the abdomen (no reported extra-abdominal cases), while MRI is the least commonly used modality (around 14.1% of cases) due to its limited role in EGB diagnosis. A systematic review on left-sided gallbladder (LSGB) conducted by Ryan *et al*, which included 112 patients, was consistent with our results that contrast-enhanced CT had the highest predictive value^[3]^.

### Association with gender and syndromes

There is no apparent correlation between gender and ectopic gallbladders (~50% in males and ~50% in females), which makes sense since the gallbladder’s development is not sex-dependent.

An ectopic gallbladder can be found alongside some syndromes. Prune Belly Syndrome (PBS) is a rare congenital disorder. PBS is characterized by a combination of three main symptoms: absence or severe underdevelopment of the abdominal wall muscles, cryptorchidism, and urinary tract abnormalities. The precise etiology of PBS is not completely comprehended, and it has been associated with many anomalies^[50]^. Gamba *et al* described its association with an anterior epigastric gallbladder^[51]^. Mirizzi syndrome occurs when the common bile duct is compressed externally by an inflamed obstructed gallbladder. It has been associated with intrahepatic gallbladder^[1]^ as well as left-sided gallbladder^[52]^. Churg–Strauss vasculitis is characterized by small and medium vessel vasculitis, hypereosinophilia, and extravascular granulomas, happening alongside severe asthma^[53]^. There has been one recorded case of a floating gallbladder accompanying Churg–Strauss syndrome; however, the anomalies don’t seem to be correlated^[54]^.

### Surgical management

Laparoscopic cholecystectomy (LC) has become the gold standard of gallbladder excision surgery, especially with rapid developments in surgical techniques and the growing experience of surgeons^[55]^. However, the lack of direct contact with the surgeon’s hands, compared with open cholecystectomy, increases the likelihood of complications such as bleeding, bile leakage, and bile duct injury, especially when the single-hole laparoscopy approach is used^[56]^. The first LC for left-sided gallbladder was reported by Schiffino *et al* in 1993^[57]^. The authors of this case indicated using the anterograde approach in cholecystectomy in order to better visualize the anatomic structures and protect the hepatic pedicle.

The “fundus-first” dissection technique, a technique used in difficult cholecystectomies where the dissection commences at the fundus of the gallbladder and progresses inferiorly towards the hilum, was found to be associated with a high incidence of bilio-vascular injuries in normal gallbladders, and yet it is not less harmful in ectopic gallbladders^[58]^.

Multiple alterations on traditional LC have been described for when LSGB is encountered. These alterations include using falciform ligament manipulation techniques (with or without division) such as the falciform lift, as well as using additional trocars or mirror image configuration^[59,60]^.

Some studies suggest combining LC and intraoperative cholangiography (IOC) to improve visualization of the biliary anatomy and reduce the damage of LC. In addition, cholecystostomy tube placement (CTP) can be used rather than LC as an alternative procedure^[59]^.

Considering the potential anatomical variations related to the condition, performing precise dissection, and ensuring a clear view of safety are essential to reducing complications during surgery. Performing antegrade dissection and intraoperative cholangiogram is recommended to reduce the risk of biliary injury during surgery^[61]^. However, Velimezis *et al* found in their single-center experience that conversion to open surgery should be the last choice only in the case of failure of the laparoscopic method or when doubting the anatomy in the region^[44]^. Velimezis *et al* firmly believe that LC can be a safe procedure even in the case of LSGB. Furthermore, when there is a doubt of an anatomical variant, intra-operative US might be useful^[44]^.

The most common cause of conversion to open surgery is the surgeon’s uncertainty about the anatomical location of the gallbladder^[62]^. Teke *et al* mentioned a case of LC of ectopic gallbladder in mesocolic position that had been converted to explorative laparotomy as they were not able to find the gallbladder^[2]^. On the other hand, Donati *et al* recommended open approach in Niemeier type I perforation (chronic perforation with fistula)^[63]^. Recently, because of the technological advances, robotic approach is becoming more common and practical due to its minimum intraoperative complications and fast recovery and resolution of symptoms^[36]^.

Recently, robotic-assisted cholecystectomy (RAC) has emerged as a promising approach for managing complex gallbladder anomalies, including ectopic gallbladder. The robotic platform provides enhanced dexterity, superior three-dimensional visualization, and improved precision, which are advantageous in cases with abnormal biliary anatomy or adhesions. Cases of RAC in left-sided gallbladder have shown favorable outcomes, including lower conversion rates and reduced complications. However, widespread adoption remains limited due to cost and access^[10]^.

Endoscopic retrograde cholangiopancreatography (ERCP) remains an important diagnostic and therapeutic tool in biliary disease. However, its effectiveness is reduced in ectopic gallbladder cases, particularly when the anatomy is distorted, such as in intrahepatic or retroplaced gallbladders. In such scenarios, successful cannulation may be challenging. Percutaneous transhepatic cholangiography (PTC) serves as a useful adjunct or alternative in cases where ERCP fails, aiding in biliary drainage or anatomical mapping prior to surgery^[47]^.

In high-risk patients who are not candidates for surgery, percutaneous cholecystostomy offers a temporizing or definitive measure. The feasibility of this technique in ectopic gallbladders depends on their accessibility, and preprocedural imaging such as contrast-enhanced CT or US is critical in planning safe access routes^[64]^.

Indocyanine green (ICG) fluorescence cholangiography can also be used for a good real-time visualization of the biliary system during laparoscopic cholecystectomy^[65]^. Since ICG is only metabolized in the liver, this technique provides excellent information about the anatomy of the biliary tree, enabling the surgeon to spot any existing anomalies and should be used if available^[65]^. ICG fluorescence cholangiography has proven its efficacy in reducing bile duct injury and overall surgical complications in cases of difficult gallbladder^[66]^. Some surgeons suggest adjusting laparoscopic ports’ sites, such as a leftward shift of all ports, which provides better maneuvering and more protection for the critical structures^[67]^.

Determining whether certain ectopic gallbladder types are more prone to gallstones or complications is very important. Unfortunately, due to the small number of cases in each subgroup, definitive trends are hard to establish.

Prior case reports have indeed highlighted that a “wandering” or floating gallbladder (suspended by a mesentery) is susceptible to torsion and even gangrene^[68]^.

Because an ectopic gallbladder can present atypically and pose diagnostic challenges, some authors recommend elective cholecystectomy for ectopic gallbladders even if the patient is asymptomatic^[44]^.

In practice, if an ectopic gallbladder is discovered incidentally (either on imaging or during surgery for an unrelated issue), we agree that the surgeon should consider removal on a case-by-case basis. Factors such as the presence of gallstones, the patient’s age and comorbidities, and any anatomical features suggesting risk (e.g. a long mesentery in a floating gallbladder) should guide the decision.

while our data alone are too limited to prove a higher risk in any specific ectopic subtype, the potential for future complications means an incidental ectopic gallbladder could merit prophylactic removal under the right circumstances. We thank the reviewer for prompting this elaboration.

### Impact on clinical practice and emergency settings

Awareness of ectopic gallbladder variants – particularly left-sided and intrahepatic types is essential for effective surgical planning and avoiding intraoperative surprises. In emergency settings, EGB may mimic other conditions, making rapid imaging-based diagnosis (contrast-enhanced CT, MRCP) and preoperative recognition vital. Procedures like ERCP or percutaneous drainage may be technically challenging, underscoring the need for early identification. This review encourages broader awareness to support preparedness and safer management in the absence of standardized surgical guidelines.

### Limitations

The consideration of ectopic gallbladder as an anatomical variant and a rare occurrence makes its diagnosis and treatment challenging. Patients present with unexplained abdominal pain, and as previously mentioned, approximately half of the patients are diagnosed during the surgical procedure. This makes the diagnosis a challenge prior to surgical intervention.

We also encountered limitations in the study design, such as the small number of patients, the fact that most of the existing results are sporadic case reports, and that some are only partially documented and lack intraoperative and postoperative data. Therefore, it is difficult to generalize our findings. However, we encourage all surgeons who encounter ectopic gallbladders in the future to document them comprehensively.

## Conclusion

Ectopic gallbladder is a rare anatomical variant that poses diagnostic and intraoperative challenges, particularly when undetected preoperatively. CT and MRCP remain the preferred imaging tools for localization and surgical planning. Laparoscopic cholecystectomy is the standard treatment, with open surgery reserved for complex or unclear cases. This review reinforces the need for increased awareness among surgeons, promoting early identification and safer, more informed surgical care despite the lack of standardized guidelines.

## Supplementary Material

## Data Availability

All data generated or analyzed during this study are included in this article.
