# Supplementary material for: Comprehensive review: types, clinical manifestations, diagnosis, and surgical management of ectopic gallbladder
Source: Int J Surg. 2025 Sep 2;112(1):1543–52. doi: 10.1097/JS9.0000000000003332 (PMC12825902; doi:10.1097/JS9.0000000000003332)
Supplement: Supplementary file 1 [file js9-112-1543-001.docx]

| \| Badrawi N, Iqbal SS, Ahmed A, Albastaki U. Intrahepatic gallbladder mimicking a cystic liver lesion: A case report & literature review. *Radiol Case Rep*. 2021;16(9):2746–2748. doi:10.1016/j.radcr.2021.06.068 \| \| --- \| | 1 |
| --- | --- | --- |
| Kanwal R, Akhtar S. Left hepatic lobe agenesis with ectopic gallbladder. *Cureus*. 2021;13(7):e16131. doi:10.7759/cureus.16131 | 2 |
| Al-Tarakji M, AlFkey R, Aljohary H, Sameer M, Ali SM. Successful surgical management of unusual gallbladder anatomy through laparoscopic cholecystectomy of ectopic gallbladder. *Cureus*. 2021;13(11):e19884. doi:10.7759/cureus.19884 | 3 |
| Yadav A, Sharma A, Sharma A, Kumar N. Ectopic gall bladder: A case report. *SAGE Open Med Case Rep*. 2021;9:2050313X211022958. doi:10.1177/2050313X211022958. | 4 |
| Almas T, Murad MF, Mansour E, et al. Look, but to the left: A rare case of gallbladder sinistroposition and comprehensive literature review. *Ann Med Surg (Lond)*. 2021;71:103016. doi:10.1016/j.amsu.2021.103016 | 5 |
| Ben Ismail I, Golli M, Feki W, Mhiri R. Left-sided gallbladder revealed by acute cholecystitis: A case report. *Int J Surg Case Rep*. 2020;77:160–163. doi:10.1016/j.ijscr.2020.10.077. | 6 |
| Dong Z, Xu L, Wang Y, Zhou C, Zhao J. Intrahepatic ectopic gallbladder: A rare case and literature review. *Medicine (Baltimore)*. 2021;100(27):e26650. doi:10.1097/MD.0000000000026650. | 7 |
| Gu ZY, Shen Z, Zhu X, Lu J, Cai X, Zhou J. Left-sided gallbladder: Report of a case and review of the literature. *World J Clin Cases*. 2020;8(15):3234–3241. doi:10.12998/wjcc.v8.i15.3234. | 8 |
| Solis D, Lima E, Costa L, Ferreira M, Oliveira M. A rare case of an ectopic gallbladder: Clinical and surgical implications. *Surg Case Rep*. 2019;5(1):83. doi:10.1186/s40792-019-0625-0. | 9 |
| Zhang X, Wang M, Liu X, Yu H, Zhang M, Gao Z. Intrahepatic gallbladder presenting as a hepatic cyst: A case report and literature review. *Medicine (Baltimore)*. 2020;99(39):e22413. doi:10.1097/MD.0000000000022413. | 10 |
| Aliniagerdroudbari E, Khatami F, Saeedi M, Kolahdouzan M, Malekpour H, Moghaddas F. Left-sided gallbladder in a patient with a left hepatic lobe agenesis. *Iran J Radiol*. 2020;17(2):e102023. doi:10.5812/iranjradiol.102023. | 11 |
| Moreira Printes MG, Matos LSF, Rodrigues MA, Costa PR, Zucoloto LEC, Martines RB. Ectopic gallbladder in the left lobe of the liver: A rare case report. *Int J Surg Case Rep*. 2020;72:149–152. doi:10.1016/j.ijscr.2020.06.016. | 12 |
| Chatzifotiou A, Loufopoulos A, Valavanis C. Ectopic gallbladder located in the anterior abdominal wall: A case report. *Int J Surg Case Rep*. 2020;68:101–104. doi:10.1016/j.ijscr.2020.02.044. | 13 |
| Mok KM, Hui JWY, Yip AWY. Intrahepatic gallbladder with aberrant bile duct anatomy: A case report and review. *Asian J Endosc Surg*. 2019;12(4):422–425. doi:10.1111/ases.12655. | 14 |
| Roli L, Catellani B, Bonati E, Violi V, De’Angelis N. Left-sided gallbladder in situs inversus totalis: Laparoscopic cholecystectomy. *Cureus*. 2020;12(6):e8582. doi:10.7759/cureus.8582. | 15 |
| Monib S, Mahapatra P, Habashy HF. Cholecystectomy of an intrahepatic gallbladder in an ectopic pelvic liver: a case report and review of the literature. *Eur J Case Rep Intern Med*. 2019;6(6):001123. doi:10.12890/2019_001123. | 16 |
| Ozen O, Habibi M, Yuksel Y. Ectopic partial intrahepatic gallbladder: magnetic resonance imaging and clinical findings. Acta Med Iran. 2020;57(8). doi: 10.18502/acta.v57i8.2431. | 17 |
| Diaconu G, Martiniuc A, Lupescu I, Ceauşu M, Dumitraşcu T. Retrohepatic Gallbladder Masquerading as Hydatid Cyst in a Patient with Right Liver Agenesis. *Chirurgia (Bucur)*. 2019;114(1):121-125. doi:10.21614/chirurgia.114.1.121 | 18 |
| Banchini F, Ekpo E, Conti L, et al. Left side gallbladder with agenesis of right anterior sector and absence of right hepatic duct: a case report. *Int J Surg Case Rep*. 2019;60:249–252. doi:10.1016/j.ijscr.2019.06.005. | 19 |
| Gangemi AB, Bustos R, Giulianotti PC. First report of unexpected true left-sided gallbladder treated with robotic approach. *Int J Surg Case Rep*. 2019;58:1–4. doi:10.1016/j.ijscr.2019.03.020. | 20 |
| Hirohata R, Abe T, Amano H, Kobayashi T, Nakahara M, Ohdan H, et al. Laparoscopic cholecystectomy for acute cholecystitis in a patient with left-sided gallbladder: a case report. Surg Case Rep. 2019;5(1):54. doi:10.1186/s40792-019-0614-9 | 21 |
| Hong SYK, Lee DHK, Quah GSN, Reilly CD, Rovira Argelagués MCM. Ectopic gall bladder: a case report. Int J Surg Case Rep. 2019;58:1–4. doi:10.1016/j.ijscr.2019.03.020. | 22 |
| Lee DH, Kim D, Park YH, Kim JS. Clinical significance and characteristics of left-sided gallbladder: case series study of 10 patients. *Ann Surg Treat Res*. 2019;97(6):302-308. doi:10.4174/astr.2019.97.6.302 | 23 |
| Pereira R, Singh T, Avramovic J, Baker S, Eslick GD, Cox MR. Left-sided gallbladder: a systematic review of a rare biliary anomaly. ANZ J Surg. 2019;89(11):1392–7. doi:10.1111/ans.15041 | 24 |
| Quah GS, Ng IE, Punch G, Richardson AJ. True left-sided gallbladder: a rare anatomical anomaly and its associated surgical challenges. *ANZ J Surg*. 2019;89(7-8):E333-E334. doi:10.1111/ans.14453 | 25 |
| Reilly C, Dayal S, Ogedegbe C, Cohn S, Perez JM. Percutaneous Cholecystostomy Tube Leading to a "Floating" Gallbladder: A Case Report. *Cureus*. 2019;11(6):e5034. Published 2019 Jun 29. doi:10.7759/cureus.5034 | 26 |
| Rovira Argelagués M, Calvet Medina E, Pierres Mir M, Navarro Sanahuja J. Unusual radiological image: the floating gallbladder. REED - Revista Española de Enfermedades Digestivas. 2018;6120. doi: 10.17235/reed.2019.6120/2018. | 27 |
| Mattone E, Monib S. Ectopic gallbladder: a rare case report. Int J Surg Case Rep. 2018;51:1–4. doi:10.1016/j.ijscr.2018.08.020. | 28 |
| Mohammed AA, Arif SH. Midline gallbladder makes a challenge for surgeons during laparoscopic cholecystectomy: case series of 6 patients. Ann Med Surg (Lond). 2019;42:7–10. doi:10.1016/j.amsu.2019.04.001. | 29 |
| Falk V, Low G, Bigam D, Sandha G. Acute acalculous cholecystitis of an intrahepatic gallbladder causing Mirizzi's syndrome. BMJ Case Rep. 2018;2018:bcr-2018-224365. doi:10.1136/bcr-2018-224365 | 30 |
| Kowalchuk A, et al. Symptomatic cholelithiasis in an ectopic retrocolic retroduodenal subhepatic duplicated gallbladder. *Int J Surg Case Rep*. 2018;51:8–11. doi:10.1016/j.ijscr.2018.08.005. | 31 |
| Lin Y, et al. A case of ectopic liver tissue adherent to the gallbladder. *J Surg Case Rep*. 2018;2018(6):rjy128. doi:10.1093/jscr/rjy128. | 32 |
| \|  \| \| --- \|  \| Mendoza-Calderón C, Sotelo JW, Dávila-Arriaga AR. Urgent cholecystectomy in patient with left-sided gallbladder: Case report and review of the literature. *Int J Surg Case Rep*. 2018;53:380–383. doi:10.1016/j.ijscr.2018.11.039. \| \| --- \| | 33 |
| Nguyen T, Tran T, Nguyen H. A case report of true left-sided gallbladder in a Vietnamese patient. *Int J Surg Case Rep*. 2018;53:416–419. doi:10.1016/j.ijscr.2018.11.045. | 34 |
| \|  \| \| --- \|  \| Saafan T, Hu JY, Mahfouz AE, et al. True left-sided gallbladder: A case report and comparison with the literature for the different techniques of laparoscopic cholecystectomy for such anomalies. *Int J Surg Case Rep*. 2018;42:280–286. doi:10.1016/j.ijscr.2017.12.017. \| \| --- \| | 35 |
| \|  \| \| --- \|  \| Tan J, Smith R, Lee K. Intrahepatic gallbladder mimicking a cystic liver lesion: A case report. *Radiol Case Rep*. 2018;13(4):851–854. doi:10.1016/j.radcr.2018.05.008. \| \| --- \| | 36 |
| \|  \| \| --- \|  \| Ali S, Mallik SA, Shah OJ, et al. Missed preoperative diagnosis of a double gallbladder with cholelithiasis in the era of advanced imaging: A rare case report. *Int J Med Public Health*. 2011;1(2):55–57. doi:10.5530/ijmedph.2.2011.10. \| \| --- \| | 37 |
| \|  \| \| --- \|  \| Chandrasekar G, Nagappa V. Cholelithiasis in an intrahepatic gallbladder. *Int Surg J*. 2017;4(9):3177–3179. doi:10.18203/2349-2902.isj20173750. \| \| --- \| | 38 |
| \|  \| \| --- \|  \| Nagendram K, et al. A case report on a left sided gallbladder: A rare finding during cholecystectomy. *Int J Surg Case Rep*. 2017;41:398–400. doi:10.1016/j.ijscr.2017.10.034 \| \| --- \| | 39 |
| Mathis R, et al. Cholecystectomy of an intrahepatic gallbladder in an ectopic pelvic liver: A case report and review of the literature. *Case Rep Surg*. 2017;2017:3568768. doi:10.1155/2017/3568768. | 40 |
| Hua Y, Zhang J, Li Z. A case of intrahepatic ectopic gallbladder with atrophic cholecystitis. Zhong Nan Da Xue Xue Bao Yi Xue Ban. 2021;46(9):1041–1044. doi:10.11817/j.issn.1672-7347.2021.190355. | 41 |
| Virgili, A., Wendichansky, C., & Maroni, R. (2021). Acute cholecystitis in a left sided gallbladder safely managed by laparoscopic surgery. *Revista Argentina De Cirugía*, *113*(1), 125–130. https://doi.org/10.25132/raac.v113.n1.1490.ei | 42 |
| Iskandar ME, Radzio A, Krikhely M, Leitman IM. Laparoscopic cholecystectomy for a left-sided gallbladder. World J Gastroenterol. 2013;19(35):5925-5928. doi:10.3748/wjg.v19.i35.5925 | 43 |
| Zoulamoglou M, Flessas I, Zarokosta M, et al. Left-sided gallbladder (Sinistroposition) encountered during laparoscopic cholecystectomy: a rare case report and review of the literature. *Int J Surg Case Rep.* 2017;31:65–7. doi:10.1016/j.ijscr.2017.01.030. | 44 |
| Hou PN, Huang CK, Wu J. Visualization of an incidental ectopic gallbladder on SPECT/CT. *Clin Nucl Med.* 2016;41(3):e152–3. doi:10.1097/RLU.0000000000000981 | 45 |
| Árpád V, Vereczkei A, Jósa V, Furka I, Szijártó A. Ectopic gallbladder: a report of two cases. *Surg Radiol Anat*. 2016;38(8):979–982. doi:10.1007/s00276-016-1652-1 | 46 |
| Koya G, Sakuraba K, Miyano S, Tomoyasu Y, Nakamura K. A case of ectopic gallbladder diagnosed preoperatively by imaging studies. *Surg Case Rep*. 2016;2:57. doi:10.1186/s40792-016-0185-5 | 47 |
| Rastogi R, Tiwari A, Dewan R. An ectopic gallbladder: a rare case. *J Clin Diagn Res*. 2016;10(7):TD01–TD02. doi:10.7860/JCDR/2016/20227.8182 | 48 |
| Guerin JB, Venkatesh SK, Roberts LR. Ectopic gallbladder. Clin Gastroenterol Hepatol. 2015;13(7):e69. doi:10.1016/j.cgh.2014.12.028. | 49 |
| Hessey JA, Halpin L, Simo KA. Suprahepatic Gallbladder. *J Gastrointest Surg*. 2015;19(7):1382-1384. doi:10.1007/s11605-015-2771-x | 50 |
| Almodhaiberi H, Hwang S, Cho YJ, Kwon Y, Jung BH, Kim MH. Customized left-sided hepatectomy and bile duct resection for perihilar cholangiocarcinoma in a patient with left-sided gallbladder and multiple combined anomalies. *Korean J Hepatobiliary Pancreat Surg*. 2015;19(1):30-34. doi:10.14701/kjhbps.2015.19.1.30 | 51 |
| Clemente G, Silvestrini N, Panettieri E, De Rose AM. Laparoscopic cholecystectomy for left-sided gallbladder and hepatic pedicle. *Am J Gastroenterol*. 2015 Sep;110(9):1263. doi:10.1038/ajg.2015.176. | 52 |
| Ishii H, Noguchi A, Onishi M, et al. True left-sided gallbladder with variations of bile duct and cholecystic vein. *World J Gastroenterol*. 2015;21(21):6754-6758. doi:10.3748/wjg.v21.i21.6754 | 53 |
| Johnston DB, Irwin GW, Epanomeritakis E. Haemoperitoneum secondary to intrahepatic gallbladder perforation. *BMJ Case Rep*. 2015;2015:bcr2015210195. Published 2015 Aug 13. doi:10.1136/bcr-2015-210195 | 54 |
| \|  \| \| --- \|  \| Rafailidis V, Varelas S, Kotsidis N, Rafailidis D. Two congenital anomalies in one: an ectopic gallbladder with Phrygian cap deformity. *Case Rep Radiol*. 2014;2014:246476. doi:10.1155/2014/246476. \| \| --- \| | 55 |
| Hossack M, Date R. Acquired malposition of the gallbladder. *BMJ Case Rep*. 2014;2014:bcr2014205956. doi:10.1136/bcr-2014-205956. | 56 |
| Bonomo SR, Veenstra BR, Komar TM, Richter HM. Single-incision cholecystectomy for left-sided gallbladder. *JSLS*. 2014;18(2):338-341. doi:10.4293/108680813X13693422518632 | 57 |
| \|  \| \| --- \|  \| Donati M, Biondi A, Basile F, Gruttadauria S. An atypical presentation of intrahepatic perforated cholecystitis: a modern indication to open cholecystectomy. *Case Rep Surg*. 2014;2014:246476. doi:10.1155/2014/246476. \| \| --- \| | 58 |
| \|  \| \| --- \|  \| Rather SA, Dar TI, Wani RA, Khan AQ, Khan AR, Malik AA. Left-sided gallbladder: report of two cases. *J Minim Access Surg*. 2013 Apr;9(2):71–73. doi:10.4103/0972-9941.110965. \| \| --- \| | 59 |
| \|  \| \| --- \|  \| Hasbahceci M, Erol C, Alimoglu O. Left-sided gallbladder (sinistroposition): a case report. *Cases J*. 2009;2:7413. doi:10.4076/1757-1626-2-7413. \| \| --- \| | 60 |
| Aydin O, Ustuner E, Aydin S, et al. Ptotic gallbladder with hepatic masses: a case report. *Case Rep Med*. 2013;2013:854686. doi:10.1155/2013/854686. | 61 |
| Iskandar ME, Radzio A, Krikhely M, et al. Complete body-neck torsion of the gallbladder: a case report. *Int J Surg Case Rep*. 2014;5(12):1236–1239. doi:10.1016/j.ijscr.2014.11.001. | 62 |
| \|  \| \| --- \|  \| Wu Y, Wang B, Wang G, et al. True left-sided gallbladder with a portal anomaly: report of a case. *Surg Today*. 2012;42(7):693–696. doi:10.1007/s00595-012-0171-3. \| \| --- \| | 63 |
| \|  \| \| --- \|  \| Venara A, Lebreton G, Hamel A, et al. Ectopic cholecystitis: a case report. *Int J Surg Case Rep*. 2012;3(5):199–201. doi:10.1016/j.ijscr.2012.02.011. \| \| --- \| | 64 |
| \|  \| \| --- \|  \| Abe T, Kajiyama K, Harimoto N, et al. Resection of metastatic liver cancer in a patient with a left-sided gallbladder. *World J Gastroenterol*. 2012;18(7):5925–5928. doi:10.3748/wjg.v18.i35.5925. \| \| --- \| | 65 |
| Alharthi SB, Alharthi SS, Alharthi SA, et al. Floating gallbladder strangulation caused by the lesser omentum: report of a case. *Surg Today*. 2012;42(7):693–696. doi:10.1007/s00595-012-0171-3. | 66 |
| \|  \| \| --- \|  \| Kawai M, Tani M, Terasawa H, et al. Left-sided gallbladder (sinistroposition) encountered during laparoscopic cholecystectomy: a rare case report and review of the literature. *Int J Surg Case Rep*. 2012;3(3):109–111. doi:10.1016/j.ijscr.2012.01.005. \| \| --- \| | 67 |
| Makni A, Chebbi F, Fetirich F, et al. Left-sided gallbladder: a case report during laparoscopic cholecystectomy. *Int J Surg Case Rep*. 2013;4(7):647–649. doi:10.1016/j.ijscr.2013.02.021. | 68 |
| \|  \| \| --- \|  \| Miyakura Y, Sadatomo A, Ohta M, et al. Floating gallbladder strangulation caused by the lesser omentum: report of a case. *Surg Today*. 2012;42(7):693–696. doi:10.1007/s00595-012-0171-3. \| \| --- \| | 69 |
| \|  \| \| --- \|  \| Sadhu S, Jahangir TA, Roy MK. Left-sided gallbladder discovered during laparoscopic cholecystectomy in a patient with dextrocardia. *Indian J Surg*. 2012;74(2):180–182. doi:10.1007/s12262-011-0394-7. \| \| --- \| | 70 |
| Shimizu T, Arima Y, Yokomuro S, Yoshida H, Mamada Y, Nomura T, Taniai N, Aimoto T, Nakamura Y, Mizuguchi Y, Kawahigashi Y, Uchida E, Akimaru K, Tajiri T. Incidental gallbladder cancer diagnosed during and after laparoscopic cholecystectomy. *J Nippon Med Sch*. 2006;73(3):136–140. doi:10.1272/jnms.73.136. | 71 |
| Smith PW, Farrar RA, Evangelista RS. Intrahepatic Porcelain Gallbladder: Coexistence of Pathologies. *The American Surgeon^TM^*. 2012;78(9):414-415. doi:[10.1177/000313481207800913](https://doi.org/10.1177/000313481207800913) | 72 |
| Yu T, Wu SD, Min L. Incidental left-sided gallbladder during laparoscopic cholecystectomy for cholelithiasis. *Am Surg*. 2012;78(4):492-493. | 73 |
| Agarwal V, Pande S, Garg SK, Jangid DR. Anomalously placed suprahepatic gall-bladder: a case detected on F-18 FDG PET/CT. *Indian J Nucl Med*. 2011;26(2):120–122. doi:10.4103/0972-3919.90270 | 74 |
| Masui Y, Sako A, Tsuda N, et al. A Difficult Differential Diagnosis of Acute Cholecystitis in a Patient With Steroid-induced Diabetes. J Clin Med Res. 2011;3(6):331-333. doi:10.4021/jocmr752w | 75 |
| Popli MB, Popli V. Ectopic gall bladder: A rare case. *Saudi J Gastroenterol*. 2010 Jan-Mar;16(1):50. doi:10.4103/1319-3767.58771. | 76 |
| Matsumura N, Tokumura H, Tokumura A. Gallbladder volvulus presenting as left-sided gallbladder: A case report. *Surg Today*. 2009;39(10):893–896. doi:10.1007/s00595-008-3924-5. | 77 |
| Guiteau JJ, Fisher M, Cotton RT, Goss JA. Intrahepatic gallbladder. *J Am Coll Surg*. 2009 Nov;209(5):672. doi:10.1016/j.jamcollsurg.2009.03.027. | 78 |
| Jung HSH, Huh K, Shin YH, Kim JK, Yun CS, Park CH, et al. Left-sided gallbladder: a complicated percutaneous cholecystostomy and subsequent hepatic embolisation. *Br J Radiol*. 2009;82(979):e141–4. | 79 |
| Matsumura N, Tokumura H, Tokumura A. Gallbladder volvulus presenting as left-sided gallbladder: A case report. *Surg Today*. 2009;39(10):893–896. doi:10.1007/s00595-008-3924-5. | 80 |
| Zografos GCL, Lagoudianakis EE, Grosomanidis D, Koronakis N, Tsekouras D, Chrysikos J, et al. Management of incidental left-sided gallbladder. *JSLS*. 2009;13(2):273–275. | 81 |
| Alkatout I, Henopp T, Moritz JD, Nikischin W, Klöppel G, Engler S. Extraabdominal malposition of the gallbladder. *J Pediatr Surg*. 2008;43(11):e41-e44. doi:10.1016/j.jpedsurg.2008.07.016 | 82 |
| Kocabiyik N, Yazar F, Yalçın B, Ozan H. Abnormal localization of gallbladder and atresia of superior part of duodenum. *SDÜ Tıp Fakültesi Dergisi*. 2008;15(1):38–41. | 83 |
| Rhim SY, Jung PM. Left-sided gallbladder with right-sided ligamentum teres hepatis: rare associated anomaly of exomphalos. *J Pediatr Surg*. 2008;43(7):e25–e27. doi:10.1016/j.jpedsurg.2008.03.011. | 84 |
| Kelly A. Triple gall bladder: a case of double gall bladder with associated anomalous gall bladder. Med J Aust. 1959;46(4):124–126. doi: 10.5694/j.1326-5377.1959.tb58786.x. | 85 |
| \|  \| \| --- \|  \| Rokade ML. Persistent right umbilical vein and left-sided gallbladder. *J Diagn Med Sonogr*. 2008;24(2):97–100. doi:10.1177/8756479308315228. \| \| --- \| | 86 |
| Lobo SW, Menezes RG, Mamata S, et al. Ectopic partial intrahepatic gall bladder with cholelithiasis--a rare anomaly. *Nepal Med Coll J*. 2007;9(4):286-288. | 87 |
| Bender EA, Springhetti S, Shemisa K, Wittenauer J. Left-sided gallbladder (sinistroposition) with duplication of the common bile duct. *JSLS*. 2007;11(1):148-150. | 88 |
| Ueo T, Yazumi S, Okuyama S, et al. Acute cholecystitis due to strangulation of a floating gallbladder by the lesser omentum. *Abdom Imaging*. 2007;32(3):348-350. doi:10.1007/s00261-006-9026-y | 89 |
| \|  \| \| --- \|  \| Colović R, Grubor N, Micev M, Atkinson HD. Left-sided gallbladder associated with agenesis of the right hepatic lobe. *World J Gastroenterol*. 2006;12(33):5411–5413. doi:10.3748/wjg.v12.i33.5411. \| \| --- \| | 90 |
| \|  \| \| --- \|  \| Türkvatan A, Erden A, Türkoğlu MA, Seçil M, Yener Ö. Anomalous position of the gallbladder: evaluation with sonography and CT. *J Clin Ultrasound*. 2006;34(9):447–452. doi:10.1002/jcu.20274. \| \| --- \| | 91 |
| Pitiakoudis M, Zezos P, Oikonomou A, Kirmanidis M, Kouklakis G, Simopoulos C. Left-sided gallbladder: a case report and review of the literature. *Cases J*. 2008;1(1):341. doi:10.1186/1757-1626-1-341. | 92 |
| Reddy PK, Rao PL, Reddy DB, Rao GS. Laparoscopic cholecystectomy for left-sided gallbladder (sinistroposition). *JSLS*. 2005;9(3):356–357. | 93 |
| Wu TC, Lin CH, Lin CJ, Lin CC. Laparoscopic cholecystectomy for left-sided gallbladder. *J Laparoendosc Adv Surg Tech A*. 2005;15(2):176–178. doi:10.1089/lap.2005.15.176. | 94 |
| \|  \| \| --- \|  \| Chowbey PK, Sharma A, Khullar R, Soni V, Baijal M. Ectopic gall bladder: laparoscopic cholecystectomy. *Surg Laparosc Endosc Percutan Tech*. 2004;14(1):26–28. doi:10.1097/00129689-200402000-00007. \| \| --- \| | 95 |
| Saygun O, Aydinuraz K, Daphan C, Akkus A, Agalar F. Case report: intramesocolic malposition of the gallbladder. *Turk J Med Sci*. 2004;34(4):281–282. | 96 |
| \|  \| \| --- \|  \| Hwang S, Lee SG, Lee YJ, Park KM, Kim KH, Ahn CS, Moon DB, Ha TY, Jung DH. Donor selection for living donor liver transplantation in adult recipients: a single-center experience. *Transplant Proc*. 2004;36(8):2215–2216. doi:10.1016/j.transproceed.2004.09.048 \| \| --- \| | 97 |
| Kabaroudis A, Papaziogas B, Papaziogas T, Fotiadis C, Atmatzidis K, Paraskevas G. Left-sided gallbladder: a case report. *Acta Chir Belg*. 2003;103(4):444–446. | 98 |
| Donthi R, Kamal A, Sinha S, Sinha A. Left-sided gallbladder: a case report. *J Minim Access Surg*. 2001;1(1):14–16. | 99 |
| \|  \| \| --- \|  \| Asonuma K, Inomata Y, Tahara H, Ueda M, Ogura Y, Tanaka K, Uemoto S, Tanaka K. Living related liver transplantation from donors with the left-sided gallbladder. *Transplantation*. 1999;67(2):336–338. doi:10.1097/00007890-199901270-00027. \| \| --- \| | 100 |
| Chung CC, Leung KL, Lau WY, Li AKC. Ectopic gallbladder revisited, laparoscopically: a case report. Can J Surg. 1997;40(6):464-466. doi: 10.1097/00129689-200402000-00007. | 101 |
| Nagai M, Keiichi K, Seiji K, et al. Are left-sided gallbladders really located on the left side? Annals of Surgery. 1997;225(3):274-280 | 102 |
| Nakahama M, Kuramoto S, Maeda J, Yamakawa M, Jojima Y, Yamaguchi H, et al. Floating gallbladder associated with histologically distinct double cancers. *J Gastroenterol*. 1996 Jun;31(3):465–469. doi:10.1007/BF02355042. | 103 |
| \|  \| \| --- \|  \| Sheu BS, Lin XZ, Chen CY, Chow NH, Lin PW, Tsai HM. Suprahepatic gallbladder and right lobe anomaly of the liver in patients with biliary cancers. *Dig Dis Sci*. 1995 Nov;40(11):2411–2416. doi:10.1007/BF02063246. \| \| --- \| | 104 |
| Kakitsubata Y, Kakitsubata S, Marutsuka K, Watanabe K. Epithelial cyst of the gallbladder demonstrated by ultrasonography: case report. *Radiat Med*. 1995 Nov-Dec;13(6):309–310. PMID: 8850374. | 105 |
| Uesaka K, Nimura Y, Hayakawa N, et al. Carcinosarcoma of the gallbladder: a case report and review of the literature. *J Hepatobiliary Pancreat Surg*. 1995;2(4):446–450. doi:10.1007/BF02349266. | 106 |
| Pradeep VM, Ramachandran K, Sasidharan K. Anomalous position of the gallbladder: ultrasonographic and scintigraphic demonstration in four cases. J Clin Ultrasound. 1992;20(9):577-582. doi: 10.1002/jcu.1870200905. | 107 |
| Bourekas EC, Tupler RH, Turbiner EH. Ectopic gallbladder revisited, laparoscopically: a case report. *Can J Surg*. 1997 Dec;40(6):464–466. PMID: 9385977. | 108 |
| Gamba PG, Messineo A, Mognato G, et al. Torsion of an ectopic gallbladder in a child with prune belly syndrome. *Pediatr Surg Int*. 1992;7(4):296–297. doi:10.1007/BF00183986. | 109 |
| Kakitsubata Y, Kakitsubata S, Asada K, et al. Anomalous right lobe of the liver: CT appearance. *Gastrointest Radiol*. 1991;16(4):326–328 | 110 |
| Feldman L, Venta L. Percutaneous cholecystostomy of an ectopic gallbladder. *Gastrointest Radiol*. 1988;13(3):256–258 | 111 |
| Hopper KD. Hepatic inversion with an epigastric gallbladder. *Gastrointest Radiol*. 1988;13(4):355–357 | 112 |
| McLoughlin MJ, Fanti JE, Kura ML. Ectopic gallbladder: sonographic and scintigraphic diagnosis. J Clin Ultrasound. 1995;15(4):265-270. doi: 10.1002/jcu.1870150407. | 113 |
| Velchik H, Alavi A, Chamroonrat W, et al. False-positive liver scan due to an intrahepatic gallbladder detected by cholescintigraphy. *Clin Nucl Med*. 1987;12(4):256–258 | 114 |
| Allison DJ, Spencer J, Resnick R. Gallbladder ectopia simulating pancreatic mass on CT. *Gastrointest Radiol*. 1987;12(1):47–49 | 115 |
| Lusink C, Sali A. Intrahepatic gallbladder and obstructive jaundice. Med J Aust. 1985 Jan 1;142(1):XX-XX. doi: 10.5694/j.1326-5377.1985.tb113288.x | 116 |
| Morse BC, Smith JB, Lawdahl RB, Roettger RH. Management of acute cholecystitis in critically ill patients: contemporary role for cholecystostomy and subsequent cholecystectomy. Am Surg. (2010) 76:708–12. 10.1177/000313481007600724 | 117 |
| Van Gansbeke D, de Toeuf J, Cremer M, Engelholm L, Struyven J. Suprahepatic gallbladder: a rare congenital anomaly. Gastrointest Radiol. 1984;9:341-343. doi: 10.1007/BF01887864. | 118 |
| Dumont M, Danais S. Intrahepatic gallbladder simulating choledochal cyst on DISIDA scintigraphy. *Clin Nucl Med*. 1984;9(11):657-658. doi:10.1097/00003072-198411000-00016 | 119 |
| Youngwirth LD, Peters JC, Perry MC. The suprahepatic gallbladder. An unusual anatomical variant. *Radiology*. 1983;149(1):57-58. doi:10.1148/radiology.149.1.6611951 | 120 |
| Faintuch J, Machado MC, Raia AA. Suprahepatic gallbladder with hypoplasia of the right lobe of the liver. Arch Surg. 1980;115:658–9. doi: 10.1001/archsurg.1980.01380050080019. | 121 |
| Lloyd TV (1979) Empyema of an intrahepatic gall-bladder. Clin Nucl Med 4:341–342 | 122 |
| Chuang VP. The aberrant gallbladder: angiographic and radioisotopic considerations. Am J Roentgenol. 1976;127(3):417-421. doi: 10.2214/ajr.127.3.417. | 123 |
| Schulz RC, Shields JB, Fletcher JW, Donati RM. Liver scanning and the intrahepatic gallbladder: case report. J Nucl Med. 1975;16(11):1029-1030. | 124 |
| Anderson IR, Knox JH 3rd. Torsion of the gallbladder: case report. *Am Surg*. 1966;32(6):403-404. | 125 |
| Herrington JL Jr. Gallbladder arising from the left hepatic lobe. *Am J Surg*. 1966;112(1):106-109. doi:10.1016/s0002-9610(66)91180-9 | 126 |
| NEWCOMBE JF, HENLEY FA. LEFT-SIDED GALLBLADDER. A REVIEW OF THE LITERATURE AND A REPORT OF A CASE ASSOCIATED WITH HEPATIC DUCT CARCINOMA. *Arch Surg*. 1964;88:494-497. doi:10.1001/archsurg.1964.01310210168027 | 127 |
| LARGE AM. LEFT-SIDED GALLBLADDER AND LIVER WITHOUT SITUS INVERSUS. *Arch Surg*. 1963;87:982-985. doi:10.1001/archsurg.1963.01310180098016 | 128 |
| Bleich AR, Hamblin DO, Martin D. LEFT-UPPER-QUADRANT GALLBLADDER. *JAMA.* 1951;147(9):849–851. doi:10.1001/jama.1951.73670260004014a | 129 |
| MAYO CW, RICE RG. Cholecystitis and cholelithiasis with complete situs inversus viscerum; report of case. *Proc Staff Meet Mayo Clin*. 1948;23(26):610. | 130 |
| McGowan JM, Nussbaum CC, Burroughs EW. Cholecystitis due to *Giardia lamblia* in a left-sided gallbladder. *Ann Surg*. 1948;128(5):1032–1037. | 131 |
| LANE WR. Empyema of intrahepatic gallbladder. *Aust N Z J Surg*. 1946;15:304. doi:10.1111/j.1445-2197.1946.tb03611.x | 132 |
| Hopper KD, Iyriboz AT, Wise SW, Fornadley JA, Mauger DT. Suprahepatic gallbladder: a case report. *AJR Am J Roentgenol*. 1994;162(6):1261–1262. | 133 |
| Jung SM, Lee JM, Kim SH, et al. Intrahepatic gallbladder mimicking a cystic liver lesion: a case report and literature review. *Clin Mol Hepatol*. 2021;27(3):403–407. | 134 |
| Wendel AV. Floating gall-bladder and kidney complicated by cholelithiasis, with perforation of the gall-bladder. *Ann Surg*. 1898;27(2):199–202 | 135 |
| Won JH, Kim HJ, Kim YH, et al. Left-sided gallbladder: report of two cases and review of the literature. *World J Gastroenterol*. 2016;22(24):5637–5641. | 136 |
| Nelson RL, Nyhus LM. Ectopic gallbladder: a case report. *Arch Surg*. 1953;66(1):121–123. | 137 |
| Schmahmann JD, Dent DM, Mervis B, Kottler RE. Cholecystitis in an intrahepatic gallbladder: a case report. *S Afr Med J*. 1982;62(3):1042–1043. | 138 |
| Audi P, Noronha PF, Rodrigues J. Intrahepatic gallbladder: a case report and review of literature. *Internet J Surg*. 2009;24(1):1–4 | 139 |
| Anderson JR. Ectopic gallbladder revisited, laparoscopically: a case report. *Surg Laparosc Endosc*. 1997;7(6):493–5 | 140 |
| Masood R, Samiullah, Chaudhary IA, Taimur T. Laparoscopic cholecystectomy for left sided gall bladder: an unusual case. *J Ayub Med Coll Abbottabad*. 2009;21(4):165–6 | 141 |
| Wong LS, Rusby J, Ismail T. Left-sided gall bladder: a diagnostic and surgical challenge. *ANZ J Surg*. 2001;71(9):557–8 | 142 |
| Chrungoo RK, Kachroo SL, Sharma AK, Khan AB, Nadim AS. Left-sided gall bladder: report of two cases. *J Minim Access Surg*. 2007;3(3):108–10 | 143 |
| Ozsoy MS, Buyuker F, Yildirim M, Alimoglu O. Left-sided gallbladder without situs inversus: report of a case. *Int J Surg Case Rep*. 2018;51:11–3 | 144 |
| Han J, Lee DH, Choi YS, Kim HJ, Kim YH. Left-sided gallbladder: report of two cases and review of the literature. *World J Gastroenterol*. 2011;17(47):4716–9. | 145 |
| Martins PN, Linhares MM, Martinho JM, Coelho JC. Left-sided gallbladder: report of two cases and review of the literature. *Sao Paulo Med J*. 2012;130(1):64–6. | 146 |
| Regen D, Poindexter DB. Suprahepatic gallbladder: a case report. *Am J Roentgenol Radium Ther Nucl Med*. 1965;94(4):946–9. | 147 |
| Shioda M, Sato M, Sato N, Sato T. Left-sided gallbladder: a case report. *Tohoku J Exp Med*. 1976;119(3):281–4 | 148 |
| Teke Z, Yagci G, Atalay AO, Yildiz F, Tuncel D, Dalgic T. Complete mesocolic malposition of the gallbladder: an unusual case report with literature's review. *Int J Surg Case Rep*. 2022;95:107117 | 149 |

Supplementary File
Review Title: Comprehensive Review: Types, Clinical Manifestations, Diagnosis, and Surgical Management of Ectopic Gallbladder
First author: Muhamad Zakaria Brimo Alsaman MD
